# The Medical Corps of the Empire State

**Published:** 1891-04

**Authors:** 


### THE MEDICAL CORPS OF THE EMPIRE STATE.

WE TAKE unusual pleasure in calling attention to the following clipping from the editorial columns of the *Buffalo Morning Express* of March 7, 1891, which will be found of interest to many of our readers at this time, and we trust all such will give its subject matter thoughtful consideration, and have speech of the same, as well, to their neighbors :

### IN THE INTEREST OF HEALTH.

Heretofore the relations existing between the people and the medical profession have been fixed by the profession itself, on the presumption that the patients exist for the physician, and that the ignorance and necessities of the people must administer to the pleasure and gain of the doctors. This relation will be radically changed after September 1st next. The State declares that on and after that date there shall be a medical profession for the good of the people. As the people know that they are comparatively ignorant of medicinal science, it is proposed that the doctors shall be kept under closer supervision and shall be duly licensed and declared competent by an official licensing board.

The Regents of the University of the State of New York will have the power to determine the qualifications and competency of our future doctors. The standard cannot be made too high. The right to practise medicine in this country is sometimes granted to men whose ignorance is simply criminal, and who would not be allowed to practise medicine in any other civilized nation of the world. When the Regents look into the matter to decide the measure of knowledge which shall be required, they need not go out of the United States for examples upon which to model their examinations. For many years the United States has maintained a medical corps, military and naval. The standard of requirements for the U. S. Medical Corps will be a safe and suitable standard for the future medical corps of New York. About 80 per cent. of the applicants for the U. S. Medical Corps are rejected as unfit, because of ignorance. Yet these applicants are all eligible to practise medicine in this State by reason of having medical diplomas from some so-called school of medicine. It may be objected that this standard is too high. This or any other standard will be considered too high by the men who are ignorant of science, yet regard themselves as possessed of the divine right to practise medicine. Every competent and self-respecting physician, however, will rejoice at the making of a high official standard. No others ought to be allowed to deal with the health and life of the people.

The New York State Board of Health reports 104,000 deaths in this State last year. Do not these figures show that the physicians of New York should only be recruited from the best material obtainable? The report shows the further significant fact that over 33,000 deaths were from specific contagious diseases. These cases ought to have been, in many instances, preventable. The matter of public health is so important that any discussion of a standard of medical proficiency lower than the highest attainable is out of the question. We have been too long accustomed to bear without protest the periodical visitations of such deadly scourges as diphtheria, typhoid fever and small-pox. No effort is too costly, no precaution too painstaking, and no skill too high for the work of preventing these epidemics.

The Board of Regents must see that the medical corps of the State of New York is composed of the best, and only the best, talent available.

Certainly the question inferentially therein propounded, What manner of a medical profession shall the early years of the twentieth century find in the Empire State? is one of the first importance, not only to every member of the medical profession, but also to every actual or potential citizen of the Commonwealth.

The position of the Journal regarding the questions of State License for physicians, a higher standard of medical efficiency, a State Medical Corps, is too well known to require restatement. From the beginning of the movement we have been in it, and from the first we have maintained that the best attainable was none too good.

Now that the discussion of what standard of proficiency the Regents of the University shall maintain is on, we want a place, and we begin with the declaration, that the tests applied to the candidates for admission to the Medical Corps of the U. S. Army or Navy are not a particle too severe for the Medical Corps of the State of New York.

Should any of our readers think this inexpedient, unwise, or impracticable, we would call their attention to the almost inexhaustible fertility and productiveness of our modern civilization. In war or in peace, in science, art, or industry, the demand is never clearly felt and plainly expressed, without the supply being forthcoming; and what our people have been doing for the last century in science and industry, our medical schools and hospitals can do in medicine.

We have perfect confidence in the situation; whatever standard of medical proficiency the Regents may exact, that degree, and better too, the schools will furnish.

But some may urge: Granted that men may be found who will take the pains to so equip themselves with a generous medical education, is it after all necessary? To this very popular question we can only cite the universal experience of every civilized community, and remark that such experience will probably be a safe guide even for our own State.

Or, it may be urged that the demands upon the skill and capa city of the Medical Corps of the United States require a higher degree of ability than is required of the general practitioner, and upon such a contention we would like to remark, that nothing could be more absurd and unintelligent.

The surgeon in the Army or Navy has under his care a well-fed and well-housed body of picked men, men selected for their soundness, men whose age exempts them from many acute and deadly diseases common to the very young and to those of advanced age, and the surgeon's care is to keep them well in the time of peace and to look after their accidents in time of war.

Now, for a moment, place this responsibility along with that which the civil practitioner has to carry, and see who has the greater need of ability, resources and efficiency. Space will not permit us to more than indicate the classes or kinds of work in civil life,—the reader can easily fill out the sketch. We would refer to the training of girls, the dangers of maternity, the care of infants, the treatment of the deformed, the management of those of evil hereditary tendency, the supervision of the public health in all of its various departments, the care of the various and varied casualties from the multitudinous wheels of industry, and the ever-present care and responsibility in the protection of communities against preventable diseases.

Surely no thinking person can for a moment maintain that in scope, in variety, in importance, the work of the military medical man can compare with the task of his brother in civil life!

Therefore, we demand, in the interests of the people, that the Regents see to it that the Medical Corps of the Empire State shall at least have as high a standard as that of the General Government or any of our sister States.

